# Integrated solutions for sustainable fall prevention in primary care, the iSOLVE project: a type 2 hybrid effectiveness-implementation design

**DOI:** 10.1186/s13012-016-0529-9

**Published:** 2017-02-07

**Authors:** Lindy Clemson, Lynette Mackenzie, Chris Roberts, Roslyn Poulos, Amy Tan, Meryl Lovarini, Cathie Sherrington, Judy M. Simpson, Karen Willis, Mary Lam, Anne Tiedemann, Dimity Pond, David Peiris, Sarah Hilmer, Sabrina Winona Pit, Kirsten Howard, Lorraine Lovitt, Fiona White

**Affiliations:** 1 Faculty of Health Sciences, The University of Sydney, Lidcombe, Australia; 2 Centre of Excellence in Population Ageing Research, Sydney, Australia; 3 Sydney Medical School - Northern, The University of Sydney, Sydney, Australia; 4 School of Public Health & Community Medicine, University of New South Wales, Sydney, Australia; 5 The George Institute for Global Health, Sydney Medical School, The University of Sydney, Sydney, Australia; 6 Sydney School of Public Health, The University of Sydney, Sydney, Australia; 7 Faculty of Health Sciences, Australian Catholic University, Melbourne, Australia; 8 School of Medicine & Public Health, University of Newcastle, Newcastle, Australia; 9 Kolling Institute of Medical Research, The University of Sydney, Sydney, Australia; 10 University Centre for Rural Health, Sydney Medical School, The University of Sydney, Sydney, Australia; 11 Clinical Excellence Commission, Sydney, Australia

## Abstract

**Background:**

Despite strong evidence giving guidance for effective fall prevention interventions in community-residing older people, there is currently no clear model for engaging general medical practitioners in fall prevention and routine use of allied health professionals in fall prevention has been slow, limiting widespread dissemination. This protocol paper outlines an implementation-effectiveness study of the Integrated Solutions for Sustainable Fall Prevention (iSOLVE) intervention which has developed integrated processes and pathways to identify older people at risk of falls and engage a whole of primary care approach to fall prevention.

**Methods/design:**

This protocol paper presents the iSOLVE implementation processes and change strategies and outlines the study design of a blended type 2 hybrid design. The study consists of a two-arm cluster randomized controlled trial in 28 general practices and recruiting 560 patients in Sydney, Australia, to evaluate effectiveness of the iSOLVE intervention in changing general practitioner fall management practices and reducing patient falls and the cost effectiveness from a healthcare funder perspective. Secondary outcomes include change in medications known to increase fall risk. We will simultaneously conduct a multi-methodology evaluation to investigate the workability and utility of the implementation intervention. The implementation evaluation includes in-depth interviews and surveys with general practitioners and allied health professionals to explore acceptability and uptake of the intervention, the coherence of the proposed changes for those in the work setting, and how to facilitate the collective action needed to implement changes in practice; social network mapping will explore professional relationships and influences on referral patterns; and, a survey of GPs in the geographical intervention zone will test diffusion of evidence-based fall prevention practices. The project works in partnership with a primary care health network, state fall prevention leaders, and a community of practice of fall prevention advocates.

**Discussion:**

The design is aimed at providing clear direction for sustainability and informing decisions about generalization of the iSOLVE intervention processes and change strategies. While challenges exist in hybrid designs, there is a potential for significant outcomes as the iSOLVE pathways project brings together practice and research to collectively solve a major national problem with implications for policy service delivery.

**Trial registration:**

Australian New Zealand Clinial Trials Registry ACTRN12615000401550

## Background

There is strong evidence that falls in community-residing older people can be prevented with clear guidance for effective interventions [1–3]. Primary care, which can include general practice, allied health services, community health, and community pharmacy, is generally the first point of contact people have with a health system. General practitioners (GP), in particular are relied on to manage the needs of older patients who are falling [4, 5]. However, there is currently no clear model for engaging GPs in fall prevention, and few older people are asked by their GP about falls or are offered interventions to prevent falls [6]. This leaves a huge gap of missed opportunity [7, 8]. Among those GPs that do address falls, few base their falls prevention practice on recognized clinical guidelines [9]. Further, the development of care plans and routine use of allied health professionals in fall prevention has been slow. This has been attributed to organizational barriers, difficulty for GPs in initiating referral and/or reimbursement processes [10], time constraints, a lack of educational materials [9], and limited understanding of what allied health professionals can offer in fall prevention [11]. While there has been research about the acceptability and uptake of care plans, there is limited research on the efficacy of multidisciplinary team approaches as an intervention to improve clinical outcomes [12].

Another major problem for evidence uptake is that successful trials of fall interventions have more often been provided within a research context. Several studies have failed to find fall prevention effects from interventions that identify older people at risk of falls in general practice followed by referral to usual care [7, 13–16]. It appears that usual care may not reflect evidence-based fall prevention. To determine why multi-factorial programs are successful or not, careful consideration needs to be given to the content of the fall prevention intervention, the process in which the intervention content is delivered and if the intervention is targeted to the appropriate group [17]. Lovarini et al.’s systematic review [18] demonstrated a lack of fall prevention research empirically testing sustainability of intervention implementation. We need to find ways of implementation in real-life contexts that are evidence-based, generalizable, and are sustainable.

A systematic review of qualitative studies investigated factors influencing the implementation of falls prevention programs. [19] This review included studies that collected information on the views of older people and health professionals. Barriers and facilitators include practical considerations (economic, access, time), adaptability of the program for the community served (social and cultural factors), and psychosocial factors (being identified as a faller, impact of falls, client preferences, the way advice is delivered and by whom). A systematic review of interventions conducting training and dissemination of evidence to healthcare professionals [20] provides evidence that training can improve implementation, but conclusions were based on six studies of which only one was a controlled trial. There was mixed support for effectiveness of changes to primary care management of falls, peer or lay-volunteer interventions, and community awareness campaigns.

Identified factors for uptake and implementation of fall prevention in primary care include accessible pathways for referral which identify people most at risk, engagement in collaborative partnerships and developing opportunities for knowledge transfer and up-skilling, thereby building capacity and ensuring better outcomes and broader population reach [9, 13]. For example, Ganz et al.’s implementation of fall prevention into a veteran’s organization [21, 22] found that the key to cultural change was collaboration and links between older people and health service providers and the development and support of healthcare organizations/systems to better deliver falls prevention interventions. Mahoney et al. [23], when implementing a community-based fall prevention program for widespread dissemination in the USA, demonstrated how evidence-based training needs to be supported by implementation guidelines for partner organizations to ensure successful adoption.

In order to test a model where general practice and allied health services are integrated and evidence-based, we conducted a small feasibility trial [24] involving two GP practices consisting of eight GPs and two private practices of physiotherapists and occupational therapists, with fall prevention interventions delivered under a Medicare rebate system (Enhanced Primary Care for Allied Health Professionals) providing partial service payment. Individual measures showed clinically important changes with excellent adherence.

### Aim and research questions

To better inform authentic sustainable population approaches to fall prevention, we have developed the Integrated Solutions for Sustainable Fall Prevention (iSOLVE) project. The aim of iSOLVE is to establish integrated processes and pathways to identify older people at risk of falls and engage a whole of primary care approach to fall prevention. We will achieve this by improving access to appropriate fall prevention interventions for older people, ensuring ongoing knowledge acquisition and sustainable action by medical practitioners and allied healthcare professionals; implementing decision-making support within general practice; and generating pathways to facilitate implementation through collaboration within a regional health network.

#### Research questions

1. The primary effectiveness research questions are to evaluate if the iSOLVE model will be effective in (i) reducing falls in patients of participating practices compared to control practices and (ii) increasing general practitioners’ engagement in fall prevention management and referral practices compared to the control GPs.
2. The primary implementation research questions will (i) investigate how the iSOLVE strategies are adopted (or not) at individual (GP and allied health professional (AHP)) practice, and primary care network levels; and (ii) identify the factors that will facilitate the embedding of the intervention in usual care, and thus its sustainability.

## Methods/design

Our study uses a type 2 blended design which places equal importance on evaluating both intervention effectiveness and implementation [25]. This design is aimed at providing clear direction for sustainability and informing decisions about generalization of the iSOLVE strategies [25]. We will test the effectiveness of the iSOLVE pathways model through a cluster randomized controlled trial to determine effectiveness for individual patients and change in GP referral behaviours. The implementation evaluation is utilizing a multi-methodology process including in-depth interviews and surveys, social network mapping, and surveying of GPs in the geographical intervention zone.

### Study setting

Partnerships are crucial in all phases of the project: development, implementation and in longer term plans for dissemination and sustainability [26]. [27] We are conducting the project in a metropolitan area and partnering initially with the Northern Sydney Medicare Local which, due to change in Federal Government policy, was replaced in 2016 by the Sydney North Primary Health Network (SNPHN), effectively doubling its geographic area. The networks have a role in educating and engaging primary care and allied health professions to enable access to effective health services. Another partner is the New South Wales (NSW) state Clinical Excellence Commission’s Fall Prevention Program which provides state-wide leadership in implementing NSW Health Falls Policy and sharing of knowledge and resources. Our final partnership is with the community of practice of fall prevention advocates. An advisory committee consisting of 14 members represents the range of allied health, community pharmacy, and primary care physicians within the district, along with consumer representation. The committee members are a highly skilled group with a broad range of perspectives on implementing fall prevention, providing support, advocacy, and informal guidance to the project.

### The iSOLVE intervention

#### Theoretical framework

Our theoretically informed iSOLVE pathways model will be integrated within one region, the Sydney North Primary Health Network (SNPHN) using the GP practice as the core focus to develop and implement effective clinical pathways by re-shaping the professional connections between potential fall service providers and the GP practice. Building decision support tools, pathways, routine referral processes, and active networks will be a way of levering evidence into practice [28]. The development has been guided by the knowledge-to-action framework (KAT) [29] which uses practical steps to implement and sustain evidence in practice: adaptation of knowledge to the local context, assessing barriers to implementation, tailoring the intervention, and evaluating outcomes. Michie et al.’s [30] behaviour change wheel provides a resource for determining key elements of behaviour change within iSOLVE such as understanding what aspects of incentives, enabling and training are key to effective functioning of our emergent model. Further, the normalization process theory (NPT) [31], considered a major theory to guide the sustainability of healthcare innovations, will frame our understanding of, and our approaches to, how new practices can be routinely used by organizations as it focuses on processes to enable new practices to become embedded. And finally, Lau et al.'s review [5] provides a useful and practical example of factors which influence change in practice in primary care.

### Components of the iSOLVE pathway model of intervention

Table 1 provides a description of the four components of the iSOLVE intervention and summarizes the planned active ingredients of these components. *Component one*: *Identifying and managing fall risk in general practice* comprises individual face-to-face training and a comprehensive set of tools to support GP’s in knowledge translation, education, and in identifying and managing fall risk. *Component two*: *Knowledge translation*, *education*, *and up-skilling the allied health local workforce* comprises evidence-based fall prevention workshops, sharing implementation strategies and opportunities for linking services to GP practices. *Component three*: *Referral pathways in primary care* outlines the role of GPs, allied health, and the SNPHN in fall prevention and the strategies used to facilitate patient pathways to fall prevention assessment and to the different intervention options. In *Component 4*: *Diffusion and dissemination*: *a guiding strategy document* will be developed to document the iSOLVE approach as a model of care for supporting regional health networks, GP practices, ambulance services, allied health, and community pharmacists to engage in integrated pathways and evidence-based effective practices to protect older people from falling.

**Table 1:** The iSOLVE intervention: planned active ingredients of iSOLVE components

Active ingredients	Description
Component 1. Identifying and managing fall risk in general practice: knowledge translation and synthesis	
1.1 Individual face-to-face training sessions	*An individual face-to-face training session* is used to educate GPs in the various components of the iSOLVE intervention including decision support tools, evidence-based interventions, potential referral pathways and fall prevention strategies. The training is based on academic detailing which is characterized by principles such as involvement of a “peer” to enable rapport and credibility, concise graphic print materials and applies social marketing principles to facilitate behavior change [58, 59].
1.2 Decision support tools and fall management tailoring. GP resources (e.g., background information/evidence, case studies, Medicare reimbursement options)	*Decision support tools and fall management tailoring* introduced in the GP training session. These decision making tools enable fall risk assessment and management and are part of a practice resource package for the GP. The decision tool and resources are adapted from a primary care resource for falls prevention, developed by the Centers for Disease Control in the USA [60]. A working group who were experts in fall prevention research, (LC, CS, ATi) drawing from the US resource and using evidence from systematic review meta-analyses of interventions [1] and of risk factors [61], developed the iSOLVE risk assessment/fall management algorithm and decision tools. The US *Stay Independent Patient Check List* and the *GP Fall Risk Assessment chart* were updated based on the iSOLVE algorithm. A new chart, *Tailoring Interventions to Fall Risk*, was developed which maps risk factors and risk factor profiles to appropriate interventions. This was based on the intervention evidence (e.g., medication review; balance, and strength training) and additionally, where intervention evidence did not exist, was based on modifiable risk factor evidence which strongly supported a guideline for practice (e.g., postural dizziness). Other iSOLVE resources include background information supporting the evidence for interventions; five case studies which each illustrate the algorithm and tailoring options and were validated by a local expert group; a detailed summary of known medications to be a risk of falling; Medicare reimbursement options for GPs; and, examples of “how to talk with patients about falls.” A summary of local allied health professionals who offer fall prevention services and who attended the workshops is provided for each GP with contact details.
1.3 GP computer systems	*GP computer systems supports*: The decision tools can be embedded into the GP systems and software by a fall prevention add-on developed for the practice software supported by the SNHN. Embedded decision tools in practice software recognize the barriers to GPs adoption of new practices and the need for speed and efficiency. The software add-on automatically creates the iSOLVE decision support tools. Once the GP completes the Fall Risk Assessment, the program produces the recommended, individualized, and tailored interventions that match their fall risk. Sample referral forms are provided. There are hard copies of all these documents, so that the whole process can be manually done if the GP chooses to or if the practice is not computerized.
1.4 Fall or fall risk alert to GP	*Fall or fall risk alert to GP:* People who report a fall in the past year or report “yes” to one of the risk questions on the *Stay Independent Fall Check list* will indicate an alert to the GP who then starts the process of assessment and management. Where practices agree a tablet device will be given to people 65 years and over by the practice nurse in the waiting room and the fall screen completed to assist in determining risk factors. If the tablet is used, this automatically sends the fall risk information to the GP’s software to speed up the process.
1.5 GP managing patient fall risk	*GP managing patient fall risk*: The GP uses the patient check list, conducts a risk assessment, and determines a tailored management plan. The management plan is generated automatically if the computer system is used. The GP may review medications and check cataracts or postural hypotension where clinically indicated. The GP also initiates appropriate referrals to local fall services (e.g., allied health and/or community exercise and/or medication review) which specify “fall prevention”.
1.6 Identifying eligible older people	*Identifying eligible older people*: People aged 65 years and over who have had a fall and or have a fall risk are identified by several processes: opportunistic presentation to the GP practice and complete *Stay Independent Patient check list* (or tablet) in the waiting room and/or are asked about falls by their GP; or, identify a fall during a 75+ annual health checks. Additionally, the falls prevention computer program initiates an annual review of fall status. These strategies are intended as routine and ongoing identification of older people who have fallen or are at risk of falling. Marketing poster and brochures are also provided for the waiting room. (Note that these approaches differ from the recruitment strategy used for patients to the trial which was adapted to ensure blinding of research assistants.)
1.7 Medication reviews	*Medication reviews*. Drawing on successful methods for reducing falls by medication review conducted by GPs in the trial conducted by Pit et al. [62]. GPs are provided with information regarding both the evidence base for reduction or ceasing medications to prevent falls and detailed lists of medications with specific fall risks. Medication reviews may also be requested using the Medicare funded Home Medicines Review by accredited pharmacists and this option is included in the *Tailoring Interventions to Fall Risk Chart*.
Component 2. Knowledge translation, education and up-skilling the allied health local workforce	
2.1 Evidence-based interactive fall prevention workshops	Educational approaches are effective in facilitating knowledge translation by AHPs [56], but active training and planning for change are needed for effective implementation and sustainability [18, 20]. Evidence-based fall prevention education workshops are offered to allied health professionals and service providers within the SNHN. These have been developed by experts within their fields and include Home Hazard and Environment interventions (LC, LM) [3]; Exercise interventions (ATi, CS) [63] and the LiFE exercise program (LC) [64], Medication Management (SH), and Foot and Ankle interventions [65].
2.2 Active planning for fall prevention implementation and sustainability	These interactive workshops comprise knowledge and skill development as well as a planning session for implementation and sustainability. Planning strategies documented by participants in each workshop form part of a developing working document shared to all workshop participants.
2.3 Linking AHPs with GPs to facilitate referrals	There is also the opportunity for AHPs to opt to be linked to GPs, thus further enhancing pathways and implementation.
Component 3. Establishing referral pathways in primary care	
3.1 Decision support tools and fall management plans	The *decision support tools and fall management plans* assist in determining the best option/s for the older person. Depending on risk assessment, this can include one or more approaches: medication review, postural hypotension assessment, referral for cataract removal, home safety assessment, community exercise programs, home-based exercise programs for higher risk patients, group-based fall exercise programs, tai chi, a community-based multifaceted fall prevention program *Stepping On* [66], or referral to a falls clinic.
3.2 Referral pathway facilitation	*Pathway facilitation.* We use a range of options to facilitate the pathway to local fall service providers, including but not limited to, the use of the Enhanced Primary Care service to encourage referral to private therapists and facilitating evidence-based fall-specific services to be provided in health and other potential care services. The aim is to provide education and knowledge translation that will up-skill and increase the fall prevention work force across the SNPHN.
3.3 Referrals to fall prevention services	*Referrals to fall prevention services*: The health network (SNPHN) and the workshops have been central to mapping local health professionals who can engage in specific interventions.
3.4 Links with ambulance services	*Ambulance services*. Fifty eight percent of older people seen by the ambulance service for a fall and not transported to hospital will fall again in the next 6 months [67]. During the development phase of the project, we will determine a process to initiate referral to GPs for fall management and follow-up. Barriers to engaging ambulance services have been investigated [68], and we will explore local options through consultation.
3.5 Network communication strategies	*Communication.* A website provides information about the project, education options, and links to state fall prevention initiatives such as *Stepping On* and the Active & Healthy website (a state directory of evidence-based fall prevention exercise options suitable for older people).
Component 4. Diffusion and dissemination of the iSOLVE model	
4.1 Development of a guiding strategy document	“The aim is to facilitate sustained implementation of evidence-based fall prevention interventions by GPs and allied health workforce. Theoretically, informed models of sustainable education and support (such as the potential for train the trainer) will be developed drawing on data gained from workshops, interviews, and observations. The Conditions for Sustainability Theory [69] and Behaviour Change Wheel Framework [30] will be used to guide this process.
	A guiding strategy document will be developed which outlines the Integrated Solutions for Sustainable Fall Prevention (iSOLVE) approach as a model of care for supporting regional health networks, GP practices, ambulance services, allied health, and community pharmacists to engage in integrated pathways and evidence-based effective practices to protect older people from falling. This will be developed by the investigators in collaboration with the partner representatives. It will be subject to extensive consultation with the key stakeholders and the Project Advisory Group.

### Effectiveness trial methodology

**Fig. 1:**
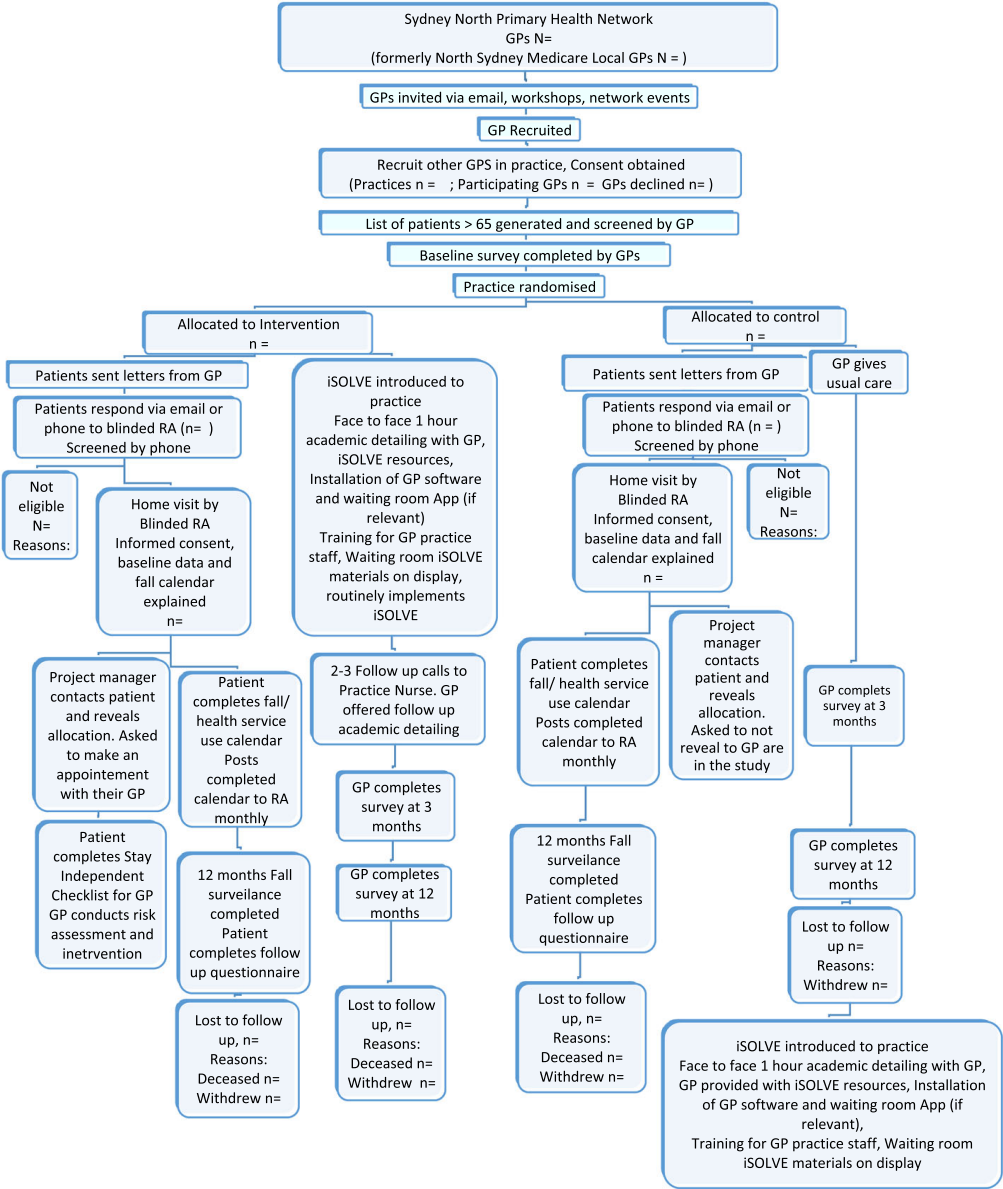
Flow of participants though cluster randomized trial

A two-arm cluster randomized controlled parallel trial is being conducted with the general practice as the unit of randomization and outcomes measured at both practice change and patient outcome levels. Cluster randomization is used in order to avoid contamination among patients of the same GP because the intervention is delivered by the GP. Randomizing GPs within the same practice to the same arm likewise avoids contamination among GPs. The trial is being conducted according to CONSORT guidelines [32]. Figure 1 provides a flow chart of participants through the cluster trial, and Table 2 summarizes the research questions and measurements. Recruitment commenced in June 2015.

**Table 2:** Research questions and evaluation

Study outcomes	Research questions	Measurement
Effectiveness	Will iSOLVE be effective in reducing falls in patients of participating practices compared to control practices?	Falls over 12 months—recorded daily on monthly calendars
	Will iSOLVE increase general practitioners’ engagement in fall prevention management and referral practices compared to the control GPs?	Pre-post survey of GPs in trial—management of fall prevention and any changes in practice (Q11: frequency of risk factor assessment, medication review, advice); referral patterns (Q12: frequency of referral and to whom); knowledge of fall prevention services.
	Will iSOLVE be effective in reducing burden of drug risk and polypharmacy in use of drugs associated with falls in patients of participating practices compared to control practices?	Drug Burden Index; Falls Risk Increasing Drugs (FRID); Changes in polypharmacy and in use of drugs associated with falls (e.g., psychotropics).
	Will iSOLVE be cost effective from a healthcare funder perspective, and expressed as an incremental cost per fall avoided?	Falls and Health care utilization—monthly calendars intervention costs—staff, training, capital costs, consumables.
	Will patients in the intervention group report significantly higher interaction with their GP about fall prevention and engagement in fall prevention activities compared to the control group?	Pre-post patient survey.
Implementation	How are the iSOLVE strategies adopted (or not) by general practitioners (GPs), by Allied health professional’s (AHP)), and by the primary care network? How does this change practice in fall prevention? How does this influence the nature of pathways for identifying older people at risk and managing fall risk within primary care?	In-depth interviews with a sample of the participating trial GPs, AHPs participating in the workshops and with key coordinating stakeholders, such as Primary Care Network staff
	What are the factors that facilitate embedding of the intervention in usual care?	In-depth interviews as above Meeting minutes, observations, and field notes. AHP planning strategies document from the workshops.
	What factors will influence sustainability of training and knowledge translation for GPs, AHPs and community pharmacist?	In-depth interviews of GPs and AHPs as above Pre- and post surveys of AHPs who participate in the workshops AHP planning strategies document from the workshops
	What is the GPs professional network in relation to fall prevention and who is influential within their network?	Relational network (who and influence) questions in survey for trial GPs. Network influence question in AHPs survey and in the ecological survey of GPs in geographical area of SNPHN
	How effective is iSOLVE dissemination across the SNPHN network?	Geographical survey of GPs in SNPHN

#### Eligibility criteria

Inclusion criteria for patients are being identified as having a fall in the past year or being worried about falling (verified using the short form of the Falls Efficacy Scale) and residing in the community. Exclusion criteria are being unable to understand the study information, an unstable medical condition, severe physical disability, or moderate to severe dementia (measured by the short portable mental status questionnaire). Inclusion criteria for general practices are that they are within the SNPHN area.

#### Outcomes

The primary outcome is the number of falls per person in 12 months. Secondary outcomes are Drug Burden Index at the patient level and GP engagement in fall prevention management and GP referral practices. Secondary outcomes are measured using surveys at baseline and 12 months for patients and baseline, 3 and 12 months for GPs in order to compare change from baseline between groups.

#### Sample size estimate

This cluster randomized trial of 560 patients from 28 GP practices is designed to have 80% power to detect as significant at the two-sided 5% level a 15% between group differences in the proportion of participants falling, from 50% in the control group to 35% in the intervention group (30% relative reduction). We have assumed 50% will fall in the control group, based on previous trials. A reduction to 35% is feasible, as a reduction of this size was found in a previous meta-analysis [33]. Assuming a cluster size of 20 participants per GP practice, an intra-cluster correlation coefficient (ICC = 0.01) and a 15% loss of patients, we require 26 clusters with a total of 520 patients. In all, 28 GP practices (560 patients) will be recruited to allow for possible loss of whole practices during the 12-month follow-up.

#### Recruitment of GP practices

GPs are invited to participate in the study by a number of approaches: promotional flyers and information distributed via the SNHN database and a database developed by the iSOLVE team by mapping GP practices using available web resources (e.g., Google™ health engines and business directories), during SNPHN events, distributed by SNPHN primary care officers, attached to the iSOLVE annual GP area survey and opportunistic such as word of mouth. For practices with multiple GPs, once one has agreed to be part of the study, others are provided with information and invited to participate.

#### Recruitment of patients

Letters are sent from the GP to people 65 years and over from their patient database. The GPs scan the list to ensure the identified patients are not living in high-care residential or hostel accommodation, do not have an unstable medical condition, are not in palliative care and are current patients. The letter invites patients to contact the project RA by phone, email, or mail if they have had a fall in the past 12 months or are worried about falling.

#### Randomization and blinding

The randomization schedule was computer-generated by a researcher at a distant site and not involved in group allocation or data collection (JS) using random permuted blocks of size 6, 4, or 2, stratified by the number of eligible patients attending the GP practice (≤80; >80). Allocation is concealed using sequentially numbered opaque envelopes. Patient lists of eligible patients are compiled by the GP practice. The allocation of practice to group occurs after the GP baseline survey, and patient lists are generated and allocation is done by a researcher not involved in practice recruitment or data collection (LC). Practices are recruited by the project manager (ATa) who also advises them of their allocation. Another researcher who is blinded to the group allocation of the GP practices contacts individual participants to explain the study, obtains signed consent, and conducts the assessments. Baseline assessments are conducted on a home visit. Patients attending the control group practices are asked not to disclose their participation in the study to their GP.

#### Intervention

The practices randomized to the intervention will receive the iSOLVE intervention, and the practices allocated to control will continue to offer usual care. Control practices will be offered the iSOLVE academic detailing, resources and software when all their patients complete the 12-month-follow-up period.

#### Measures and data collection

Patient baseline data for control and intervention patients will include history of falls and fall injuries in the past 12 months, age, hospitalization in the past year, current medications, medication dosage and frequency, and comorbidities using the Functional Comorbidity Index [34]. Patients are asked to record falls using a prospective falls calendar to be completed on a daily basis and returned by mail monthly. Fall calendars also prompt patients to record the circumstances of a fall, whether any injuries occurred and whether any medical help was sought as a result of the fall. If a person does not return their fall calendar, they are telephoned to determine whether they have fallen. A 12-month follow-up survey ascertains patient engagement in fall prevention interventions or activities over the past year, interaction with GP about fall prevention, current medications including dosage and frequency. Medication survey questions will also generate data to determine prevalence and change over 12 months in medication exposures that have been associated with increased risk of falls with Drug Burden Index [35] as the major secondary measure. Other medication data will include polypharmacy, falls risk-increasing drugs [36, 37], and use of psychotropics (antipsychotics, antidepressants, and other mood stabilizers, benzodiazepines, and z-drugs) and other drug classes of interest.

The GPs complete a survey at baseline, 3 and 12 months, comprising a combination of open-ended and Likert scale response questions. The questions cover beliefs about fall prevention, knowledge of local fall prevention services, management of fall prevention and any changes in practice, and referral patterns.

#### Data analysis plan

Data analysis will be on an intention-to-treat basis. For the primary analysis of falls, negative binomial regression will be used to compare the total number of falls per person in the two groups, using an offset for exposure time and accounting for clustering. GP engagement in fall prevention management and GP referral practices will be assessed using questions 11 and 12, respectively, of the 12-month GP survey and corresponding questions on the baseline survey. For management, a score from 0 to 3 will be allocated to each of the first three items and summed to give a total out of 9. The total score for each GP at 12 months will be compared between groups using regression analysis with baseline score as a covariate and adjusting for clustering by GP practice. For referral practice, the number of types of practitioners (out of 13 possible) to whom a GP sometimes or often refers older patients at risk of falling will be counted. The change from baseline for each GP will be compared between groups using Poisson regression, adjusting for clustering by GP practice. Change in Drug Burden Index will be calculated for each patient and compared between groups using regression analysis, controlling for any baseline differences in risk factors (age, gender, comorbidities, hospitalization in past year, and use of walking aid) and accounting for clustering.

#### Cost-effectiveness study

A healthcare funder perspective will be used for the economic evaluation. Health outcomes will be measured in terms of falls prevented. Costs will include healthcare utilization collected by monthly calendars in intervention and control participants and intervention costs (including staff, training, capital costs, and consumables). Mean costs and the mean health outcomes in each trial arm will be calculated. The incremental cost-effectiveness ratio of the intervention compared to control group will be calculated as the cost per fall prevented; results will be plotted on a cost-effectiveness plane. Bootstrapping will be used to estimate a distribution around costs and health outcomes and to calculate the CIs around the incremental cost-effectiveness ratios. One-way sensitivity analysis will be conducted around key variables and assumptions; a probabilistic sensitivity analysis will be used to examine joint uncertainty in all parameters. A cost-effectiveness acceptability curve will be plotted to provide information about the probability that the intervention is cost-effective, given decision makers’ willingness to pay for each fall prevented.

### Multi-methodology implementation evaluation

While intervention researchers ask “does the proposed intervention” work, process evaluation is required to understand “how” it works [38]. This pragmatic approach using multi-methodology aims to understand fall prevention within the “real-world” setting [39], in this case, medical and allied health practice. The evaluation will also enable a deeper understanding of the coordinating role that the Primary Care Network can play in this area. In developing an intervention within an existing set of processes, referrals, and relationships (the GP and allied health settings), Normalization Process Theory (NPT) [31] can enable identification of facilitation factors. NPT focuses on the “work” of the intervention—and how changes in the work that is required to identify and refer people at risk of falls can become routinized into the everyday practice of the GP. NPT provides a framework for designing the inquiry and framing the interpretation of data.

#### In-depth interviews and surveys

The process of understanding implementation of the trial is iterative, seeking explanatory data at all layers, and over the entire time, of the intervention. Initial process evaluation will involve gathering data synchronistically with the intended intervention. Data will include surveys, in-depth interviews, observations and field notes, and documentation of meetings. Guided by NPT, we will examine the “coherence” of the proposed change for those in the work setting and how to facilitate the “collective action” needed to implement changes in practice, as follows:

- General practices. Data will comprise documentation of the academic detailing process and in-depth interviews with a sample of GPs and practice nurses participating in the intervention cluster. The aim is to elucidate how they perceive fall prevention as an issue for their patients; the barriers and facilitators to falls identification and to implementing fall management and referral practices; and how the iSOLVE resources are used (or not) and the perceived value of the education package and resources to changing practice.
- Allied Health Professionals (AHPs). AHPs are recruited to the workshops primarily thorough the SNHN website and newsletter. Other strategies have included professional association websites and interest groups, a Google™ search of service directories and service providers identified from GPs. AHPs are eligible if they are located in the geographical area of the SNHN, provide community services, provide or intend to provide fall prevention services to older people, and are able to receive GP referrals. A pre-, 3-, and 12-month post-workshop survey is administered following AHP education sessions and in-depth interviews conducted with a sample of AHPs participating in the workshops. The AHP implementation planning strategies compiled from data collected at the workshops will be used as an additional data source. The surveys include a combination of open-ended and Likert scale response questions; practice change, frequency of assessment and interventions related to fall prevention, referral patterns (to/from), type of services offered, and level of confidence in related skills. Demographic questions record profession, service sector, and nature of employment status. The interviews will ascertain the views of AHPs on any changes (or not) in the referral process; how effective the referral process is, and the factors affecting adoption and implementation of the intervention.

Interviews will also be conducted with key coordinating stakeholders, such as Primary Care Network staff. The views of these stakeholders will provide additional insights into the factors that may affect uptake of the intervention.

#### Survey across geographical area

A survey will be used to evaluate GP referral patterns and practices as markers of uptake and implementation across the intervention region, that is, the SHPHN. The survey comprises the same questions as in the trial survey for participating GPs Trends in program perceived efficacy, adoption, and implementation [40, 41] will be monitored over the 5 years of the program. The survey is being distributed annually to all GPs via the SNPHN mail lists in electronic format. As response rates are usually low for GPs [4], we are also randomly selecting 10% of all GPs in the region and mailing them a hard copy of the survey with pen. The random sample was selected using a computer-generated random permutation of integers.

#### Social network mapping

Relational data relating to the GPs professional network is being collected within the GP surveys as part of the cluster randomized trial. A question about who is influential is also included in the AHP survey and the area GP survey. Social network analysis is emerging as a potential tool to evaluate networks in healthcare settings [42, 43]. The network of roles from whom GPs develop their knowledge base in falls prevention will be visualized using an alluvial map generator. We will explore who in their social or professional networks have influenced their knowledge and learning of fall prevention and the depth of such influence, the people who they see as important in providing fall prevention services and if there are associations between these relationships and GP fall management and referral practices. An alluvial map can show how the impact of differing professional roles can change between, before, and after the academic detailing intervention [44].

The social network analysis of personal relationships with professionals who provide a clinical service to falls patients will first describe the structure of each network of referral relations. Second, it will explore the roles of other professionals relevant to their contribution to the GPs management of patients who had or are at risk of falls. In the second stage, we will quantify the contribution of relational processes that may have contributed to the structure of referral networks that we observed using exponential random graph models (ERGMs) and model the probability of a referral relationship formation. ERGMs provide statistical tests to assess if these effects are significantly different from zero [45]. Analogous to odds ratio in logistic regression models, model coefficients are interpreted as additive factors on the log odds of forming a relationship around referral. Significant and positive coefficients indicate higher log odds of forming a relation in accordance with the underlying process; negative coefficients make it less likely. The goodness of fit of each of these models will be assessed, following the procedures in Hunter et al. [46].

#### Implementation data analysis: surveys, observation data, and interviews

The information gathered will be used as indicative and descriptive information to inform further dissemination. Responses to survey data will be coded and compared within group and over time. Data will be explored using descriptive and graphical methods and, where possible, measure change in knowledge or practice using independent sample t tests and multinomial logistic regression. Documentation relating to the intervention (e.g., minutes of meetings, discussions with stakeholders) will be examined using content analysis. Analysis will focus on the “work of the intervention” [47]—how doing the work, concerns raised about the work, and how responsibility for falls prevention strategies is distributed across different groups. All interviews will be audio-recorded and transcribed verbatim. Thematic analysis (developing codes) will identify patterns within and across the study groups [48]. A combination of inductive and deductive coding will be used—coding will commence with examination of barriers and facilitators, but will be open to unexpected findings that may contribute to these. Data analysis will examine similarities and differences within and between stakeholder and participant groups and changes evident at different time points in the intervention [49].

## Discussion

The fundamental goal of this research project is to achieve dissemination and planned sustainability of an applied model of delivery integrated processes and pathways to identify older people at risk of falls and engage a whole of primary care approach to fall prevention. The longer term aim is a model that could be used for widespread dissemination. Historically, there have been a number of challenges in implementing prevention programs into primary health. Over the past decade, there have been calls for improved detection of early fall risk, awareness and access to services, and strengthening pathways between referrers and service providers [50].

The advantages of a blended effectiveness-implementation design is that it uses mixed-methods which are designed to evaluate both content and context as a way of accelerating research into practice, in particular by avoiding lengthy step-wise approaches [51, 52]. It also offers a way of optimizing our knowledge and understanding as there is a blending and interaction of both intervention and implementation, and it provides opportunities for triangulation and richly validated findings [53].

At the same time, this approach raises a number of methodological challenges [25]. While core features of design rigor in cluster randomized trials are being adhered to [32], this is a pragmatic trial in which the intervention is being delivered in a real-world setting. We therefore have less control both of intervention fidelity for GPs and also of whether and how the recruited patients follow through with any GP-advised strategies, recommendations, or referrals. Given resources in a hybrid design need to cover a variety of expertise and personnel [25] and the fact that we minimized patient secondary assessment compared to what would be usual in an effectiveness/explanatory trial, there is a limited opportunity to capture, for example, quantitative adherence data and validated measures of participant engagement. Contamination may occur as allied health professionals who attended workshops could see control patients referred to them for other reasons or from other sources, as they have been encouraged to identify people who have fallen or at risk of falls, combined with their new skills to implement fall prevention. There are the usual generalization issues related to cluster randomized trials in defining how included practices (and patients) might differ from those not included [54]. Initial recruitment via newsletters may have included those most keen, and ongoing recruitment has been expanded to include multiple methods. Despite this, so far, we have experienced a high response rate and interest by GP practice indicating fall prevention is a topic of interest to many GPs. While threats to external validity may be heightened in this blended design, we are applying robust trial rigour, will review self-report of patient engagement in fall prevention over the follow-up period, and active dissemination of the GP resources and processes throughout the SNHN will not occur until completion of the cluster randomized trial.

As an implementation project, the context of one geographical area may well be different from others, such as rural versus metropolitan, thus introducing an effect modification. Willis et al. [55] warn how intentions for embedded strategies into existing services can be hindered by a reliance on material resources for continuation rather than embedding the processes based on key features for sustainability. Further, we know that training is a crucial and necessary first step in the process of change [56] but much more needs to be understood about how change occurs and what planning is needed [57]. Our expectation is that the qualitative analysis approach using Normalization Process Theory will give the necessary depth of understanding to the contextual issues related to implementation and sustainability. This should enable us to place appropriate emphasis on the lessons we will learn, what the key drivers for change are and what can be generalized, or what will need further research.

While challenges exist, there is potential for significant outcomes as the iSOLVE pathways project brings together practice and research to collectively solve a major national problem with implications for policy service delivery. The project outcomes will be a multi-disciplinary pathway model for fall prevention that will have been tested empirically in an urban area. It will provide evidence of resource need and allocation, and feasible processes and guidelines for fall identification and prevention in general practice and community-based healthcare practices. Practical outcomes include training modules and processes as well as a model for organizational and system support needs for implementation and sustainability of GP and allied health evidence-based practice in falls prevention. Partnerships with regional, state, and professional organizations provide opportunities for widespread dissemination, leadership, and diffusion.
